# Metabolic recovery after weight loss surgery is reflected in serum microRNAs

**DOI:** 10.1136/bmjdrc-2020-001441

**Published:** 2020-10-28

**Authors:** Susana Sangiao-Alvarellos, Konstantinos Theofilatos, Temo Barwari, Clemens Gutmann, Kaloyan Takov, Bhawana Singh, Paula Juiz-Valiña, Bárbara María Varela-Rodríguez, Elena Outeiriño-Blanco, Elisa Duregotti, Anna Zampetaki, Lukas Lunger, Christoph Ebenbichler, Herbert Tilg, María Jesús García-Brao, Peter Willeit, Enrique Mena, Stefan Kiechl, Fernando Cordido, Manuel Mayr

**Affiliations:** 1 King’s British Heart Foundation Centre, School of Cardiovascular Medicine and Sciences, King’s College London, London, UK; 2 Endocrine, Nutritional and Metabolic Diseases Group, Department of Physiotherapy, Medicine and Biomedical Sciences, Faculty of Health Sciences, University of A Coruña, A Coruña, Spain; 3 Instituto de Investigación Biomédica de A Coruña (INIBIC), A Coruña, Spain; 4 Department of Endocrinology, A Coruña University Hospital, A Coruña, Spain; 5 Department for Internal Medicine I, Medizinische Universität Innsbruck, Innsbruck, Austria; 6 Department of Digestive and General Surgery, A Coruña University Hospital, A Coruña, Spain; 7 Department of Public Health and Primary Care, University of Cambridge, Cambridge, UK; 8 Department of Neurology, Medical University of Innsbruck, Innsbruck, Austria

**Keywords:** biomarkers, obesity, morbid, adipose tissue, liver

## Abstract

**Introduction:**

Bariatric surgery offers the most effective treatment for obesity, ameliorating or even reverting associated metabolic disorders, such as type 2 diabetes. We sought to determine the effects of bariatric surgery on circulating microRNAs (miRNAs) that have been implicated in the metabolic cross talk between the liver and adipose tissue.

**Research design and methods:**

We measured 30 miRNAs in 155 morbidly obese patients and 47 controls and defined associations between miRNAs and metabolic parameters. Patients were followed up for 12 months after bariatric surgery. Key findings were replicated in a separate cohort of bariatric surgery patients with up to 18 months of follow-up.

**Results:**

Higher circulating levels of liver-related miRNAs, such as miR-122, miR-885-5 p or miR-192 were observed in morbidly obese patients. The levels of these miRNAs were positively correlated with body mass index, percentage fat mass, blood glucose levels and liver transaminases. Elevated levels of circulating liver-derived miRNAs were reversed to levels of non-obese controls within 3 months after bariatric surgery. In contrast, putative adipose tissue-derived miRNAs remained unchanged (miR-99b) or increased (miR-221, miR-222) after bariatric surgery, suggesting a minor contribution of white adipose tissue to circulating miRNA levels. Circulating levels of liver-derived miRNAs normalized along with the endocrine and metabolic recovery of bariatric surgery, independent of the fat percentage reduction.

**Conclusions:**

Since liver miRNAs play a crucial role in the regulation of hepatic biochemical processes, future studies are warranted to assess whether they may serve as determinants or mediators of metabolic risk in morbidly obese patients.

**Significance of this study**

**What is already known about this subject?**

- Lipid and glucose homeostasis are controlled by a cross talk between the liver and adipose tissue. The molecular mechanisms of this cross talk remain unknown.
- Bariatric surgery is an effective treatment for obesity. It is currently unknown how it affects the liver–adipose tissue cross talk and the levels of microRNAs (miRNAs) in the circulation.

**What are the new findings?**

- Morbidly obese patients have elevated levels of circulating liver-derived but not adipose tissue-derived miRNAs. Liver-derived miRNAs levels positively correlate with body mass index, fat mass percentage and glucose levels.
- Bariatric surgery in obese patients restores the liver-derived miRNAs in serum to healthy levels.
- Circulating levels of adipose tissue-derived miRNAs are not reduced after bariatric surgery, despite a significant weight loss. This refutes the hypothesis for a major contribution of adipose tissue to the circulating miRNA pool.

**How might these results change the focus of research or clinical practice?**

- Further studies on the liver–adipose tissue cross talk are warranted with a focus on liver-derived miRNAs.
- Liver-derived miRNAs may be explored as biomarkers for or drivers of metabolic syndrome in the setting of obesity.

## Introduction

Obesity is a chronic disease and widely recognized as the largest and fastest growing public health problem in the developed and developing world. The etiology of obesity is multifactorial but unhealthy human behavior, an obesogenic environment and inadequate healthcare advice are major contributors to the unprecedented increase in global overweight and obesity levels.^1^ Obesity contributes to the onset of metabolic disorders such as type 2 diabetes mellitus and non-alcoholic fatty liver disease (NAFLD).^2^ Although epidemiological correlations between obesity and insulin resistance in type 2 diabetes, NAFLD and metabolic syndrome are established, the cellular and molecular mechanisms that link obesity with metabolic disease remain less well explored.^2 3^

MicroRNAs (miRNAs, miR) are small non-coding RNAs that regulate gene expression at the post-transcriptional level.^4^ Circulating miRNAs are packaged in high-density lipoproteins,^5^ in protein complexes with argonaute 2^6^ and other RNA-binding proteins^7^ as well as in extracellular vesicles (EVs), including exosomes.^8^ Eukaryotic cells shed intact microvesicles from the cellular membrane and exosomes via the endosomal pathway.^9^ The composition and content of EVs vary depending on the parent cell from which they originate, as well as the cellular processes triggering their formation. It is proposed that exosomal miRNAs may be delivered to the recipient cells and participate in intercellular communication.^10 11^

There is a well-established cross talk between adipose tissue and the liver in terms of both lipid and glucose homeostasis. Recently, Thomou *et al* suggested that the principal source of exosomal miRNAs in the circulation is the adipose tissue and that adipose-derived exosomal miRNAs can regulate hepatic gene expression.^12^ Furthermore, Zhao *et al* demonstrated that the levels of several EV miRNAs in the plasma of patients with NAFLD are positively correlated with body mass index (BMI), and these miRNAs enhance adipocyte lipid accumulation.^13^

So far, bariatric surgery offers the most effective and durable treatment for obesity ameliorating or even resolving comorbidities such as type 2 diabetes.^3 14 15^ It has been demonstrated in several randomized controlled trials that bariatric surgery achieves better glycaemic control and a higher rate of type 2 diabetes remission than the exclusive use of medication.^16^ Some studies have investigated the effects of obesity and bariatric surgery on the pattern of both circulating and adipose tissue miRNAs.^17–21^ However, the beneficial potential of bariatric surgery on circulating miRNAs remains unclear. The first miRNA which was found to be related to metabolic control was miR-122. It constitutes 70% of the total miRNA content in the liver, plays a central role in lipid metabolism and was reported to be secreted in exosomes.^22 23^ Our previous findings demonstrated a strong association of circulating liver-derived miR-122 levels with the development of metabolic syndrome and type 2 diabetes.^24^ This association remained significant after multivariable adjustment, including BMI, waist–hip ratio and insulin resistance.^24^ It has been suggested that elevated levels of circulating miR-122 are reduced with weight loss and can be a marker of a harmful metabolic status.^25^

The objective of this study was to compare circulating levels of miRNAs in morbidly obese patients and healthy volunteers. Moreover, we analyzed miRNA levels in obese patients after undergoing bariatric surgery to establish its effects on circulating miRNAs.

## Research design and methods

### Patients and sample collection

Serum samples were collected from healthy, non-obese patients (controls), and morbidly obese patients before and after they had undergone bariatric surgery, either Roux-en-Y gastric bypass (RYGB) or sleeve gastrectomy. For the bariatric surgery group in the discovery cohort from A Coruña (Spain), serum samples were taken on the day of bariatric surgery (n=155, T=0) and at various intervals after surgery. The follow-up time point at which the measurements were taken varied for different patients and was 3 months (n=29, T=3), 6 months (n=27, T=6) or 12 months (n=27, T=12) after bariatric surgery. For the validation cohort from Innsbruck (North Tyrol, Austria), stored serial serum samples were available from bariatric surgery patients with a BMI >40 kg/m^2^ before surgery (n=33, T=0) and 12 months (n=14, T=12) or 18 months (n=19, T=18) after surgery, respectively. Consistent with the discovery cohort, the validation cohort also had different follow-up time points for different patients.

### Quantitative real-time PCR

Total RNA from murine serum (100 µL) was isolated with the miRNeasy Serum/Plasma Kit (Qiagen) according to the manufacturer’s instructions. Reverse transcription was then performed with the miRCURY LNA RT kit (Exiqon, catalog no: 339340) according to the manufacturer’s protocol. Real-time PCR was performed using the miRCURY Ready-to-Use PCR, Human panel I+II V1.M platform (Exiqon) on a ViiA 7 Real-Time PCR System with 384-well block. Relative miRNA expression was determined by the comparative cycle threshold (ΔΔCq) method by using the exogenous *Caenorhabditis elegans* spike-in control (*cel*-miR-39) for ΔCq and a calibrator consisting of an RNA pool of all samples for ΔΔCq.

Total RNA from patient serum (100 µL) was isolated with the miRNeasy Serum/Plasma Kit (Qiagen) according to the manufacturer’s instructions. RNA quality was determined by spectrophotometry in an ND-1000 NANODROP 385 spectrophotometer (Thermo-Scientific). Three microliters of the extracted RNA was reverse transcribed using Megaplex RT Primers, Human Pool Set v3.0 and TaqMan MicroRNA Reverse Transcription Kit (Applied Biosystems). Prior to quantitation by real-time PCR, 1 µL of undiluted RT product was preamplified using Megaplex PreAmp Primers along with the TaqMan PreAmp Master Mix (Applied Biosystems). After dilution of the sample, real-time PCR was performed using TaqMan Universal PCR Master Mix, No AmpErase UNG and specific primers to each miRNA on a ViiA 7 Real-Time PCR System with 384-well block. Relative miRNA expression was determined by the comparative ΔΔCq method by normalizing either to the *C. elegans* spike-in control (*cel*-miR-39) or to a global Cq average of 30 miRNAs: let-7b, miR-122-5p, miR-125a-5p, miR-130a-3p, miR-140-5p, miR-143-3p, miR-145-5p, miR-148a-3p, miR-148b-3p, miR-150-5p, miR-191-5p miR-192-5p, miR-19b-3p, miR-200a-3p, miR-20b-5p, miR-21-5p, miR-210-3p, miR-221-3p, miR-222-3p, miR-223-3p, miR-26a-5p, miR-27b-3p, miR-29a-3p, miR-30b-5p, miR-324-5p, miR-486-5p miR-375-3p, miR-574-3p, miR-885-5p and miR-99b-5p.

### Lep^ob^ mice

All procedures were performed in accordance with the Guidance on the Operation of the Animals (Scientific Procedures) Act 1986 (UK) with ethical approval obtained from the local ethics committee (PPL: 70/8944 to Professor Qingbo Xu). To determine whether miRNA profiles identified in bariatric patients also apply to hyperglycemic animals, we quantified miRNA levels in serum of obese mice. For this purpose, male Lep^ob^ mice aged 8–12 weeks (n=15, previously known as ob/ob) were purchased from Jackson Laboratories. Male C57BL/6J mice (n=15) were used as a control. Mice were kept on chow diet. A total of 1 mL of blood was harvested from each mouse at 14–15 weeks of age, and serum was isolated following clotting and centrifugation at 1200 g for 20 min at 4°C. Serum samples were aliquoted and stored at −80°C. All experiments were performed according to protocols approved by the Institutional Committee for Use and Care of Laboratory Animals.

### Separation of EVs from murine serum

A differential centrifugation-based protocol was used to separate small EVs (sEVs), large EVs (lEVs) and supernatant from mouse serum (wild type vs Lep^ob^ mice on C57BL/6J background, all mice aged 14–15 weeks, male and on normal diet).^26^ 100 µL of serum were diluted to 500 µL with phosphate-buffered saline (PBS) and centrifuged at 10 000 g for 30 min at 4°C. The pellet was resuspended in 100 µL PBS (=lEVs), while the supernatant was diluted to 1 mL with PBS and ultracentrifuged at 1 00 000 g for 60 min at 4°C (Beckman Coulter Optima Max ultracentrifuge with TLA-55 rotor (k-Factor 89.5) and 9.5×38 mm Beckman Coulter Microcentrifuge Polypropylene Tubes). The supernatant was collected, and the pellet was resuspended in 1 mL PBS. Ultracentrifugation was then repeated using the same settings, and the supernatant was collected and added to the supernatant obtained from the previous step to a total volume of 2 mL (EV-depleted supernatant). The pellet was resuspended in 100 µL PBS (=sEVs). As expected, dot blot analysis confirmed that syntenin-1, a marker of sEVs, was enriched in the sEV isolates, while apolipoprotein B (ApoB), the integral membrane protein of APOB-containing lipoproteins, was depleted of sEV and lEV samples in comparison to the EV-depleted fraction (online supplemental figure 1).

10.1136/bmjdrc-2020-001441.supp1 Supplementary data

### Data analysis

Relative quantification values of miRNAs and clinical variables for both discovery and validation cohorts were first filtered based on a missing value criterion. Specifically, miRNAs and clinical variables with more than 50% missing values were removed from the dataset and not considered for further analysis. For example, for T=3 C reactive protein with 76% missing values, and ApoA1 and ApoB with 83% missing values were not considered for further analysis. The miRNAs and clinical variables with less than 50% missing values were imputed using the KNN Impute algorithm.^27^ The imputation methodology was run separately for the different groups of the study. For example, for T=0 ApoA1 with 21% missing values was imputed. The limma package^28^ was used to compare between different phenotypes using the empirical Bayes algorithm^29^ and correcting for selected covariates. Paired analysis, ie, before and after surgery, was undertaken with correction for statin use to factor the change in medications of some patients after surgery. For control (healthy) and obese patients (ie before surgery, T=0), unpaired analysis was conducted. Unpaired analyses between healthy and obese participants were corrected for age, sex and statins. In all comparisons, the initial P values were adjusted for multiple testing using Benjamini-Hochberg method^30^ and a threshold of 0.05 was used for the adjusted P values to infer statistically significant changes. Spearman correlation was used for studying correlation patterns and continuous variables. Point-biserial correlation^31^ was used for studying correlation patterns between miRNAs and categorical variables (such as sex and type 2 diabetes). Linear regression analysis was performed to associate proteins with miR-122, and to relate miRNA changes after surgery with alterations in glucose, weight and liver transaminases, considering age, sex and statins as covariates and scaling values to zero means and SD of 1. All figures were generated with R programming environment and GraphPad software.

## Results

### Differences in circulating miRNAs between obese patients and controls

**Figure 1:**
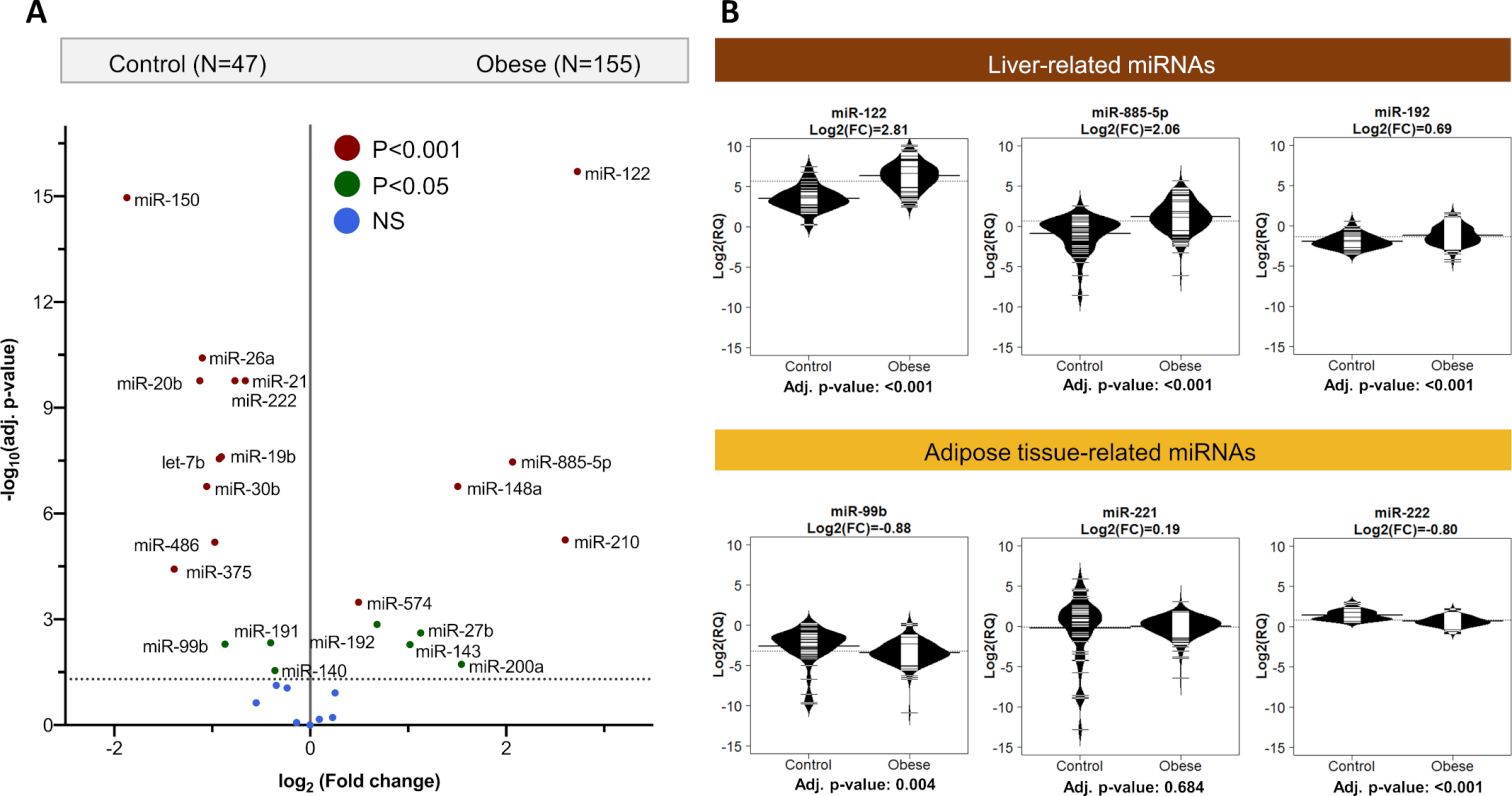
(A) Serum microRNAs (miRNAs) in obese patients and healthy controls. Volcano plot for differences in serum miRNAs between control (n=47) and obese patients (n=155) with standardized miRNA concentrations to the global Cq average. Statistical comparison was conducted using the empirical Bayes method of the limma package. P-values were corrected for multiple testing and are shown in blue, green, maroon for the different p-value ranges. Liver-specific miR-122 was among the miRNAs with the most elevated serum levels in obese patients compared with controls. (B) Bean plots for miRNAs normalized to the global Cq average. Bean plots showing elevated serum levels of liver-related (brown color) miR-122, miR-885-5 p and miR-192 in obesity. The serum levels of putative adipose tissue-related miRNAs (yellow color), such as miR-99b, miR-221 and miR-222, were not elevated in obese patients. FC, fold change; NS, not significant.

We analyzed serum samples from morbidly obese patients before bariatric surgery (n=155) and from healthy participants with normal BMI as controls (n=47). The clinical characteristics of all participants of this discovery cohort are shown in online supplemental table 1. A panel of 30 circulating miRNAs previously associated with the liver or adipose tissue as well as unrelated blood cell-derived miRNAs were selected for analysis by quantitative PCR. The most pronounced rise in morbidly obese patients was observed for miR-122, miR-885-5p, miR-148a and miR-210 (log_2_ fold change (FC) >1.5 and p<0.001). Levels of miR-150 were markedly decreased in obese patients compared with control individuals with normal BMI (figure 1A). The key findings based on standardized miRNA concentrations to the global Cq average were replicated using an exogenous spike-in miRNA, *cel-*miR-39, for normalization (online supplemental figure 2). While miR-122 and liver-related miRNAs were among the most altered miRNAs, putative adipose tissue-derived miRNAs,^12 32 33^ that is, miR-99b, miR-221 and miR-222, were detected at high levels in healthy human subcutaneous adipose tissue (data not shown) but were not elevated in serum of morbidly obese patients (figure 1B). Using *cel-*miR-39 as an alternative normalisation strategy, miR-221 showed an increase (log_2_ (FC)=1.3, p=0.02), but it was relatively small in comparison to the liver-specific miR-122 (log_2_ (FC)=3.8, p<0.001) and liver-related miR-885-5p (log_2_ (FC)=3.1, p<0.001) (online supplemental figure 3, online supplemental table 2).

**Figure 2:**
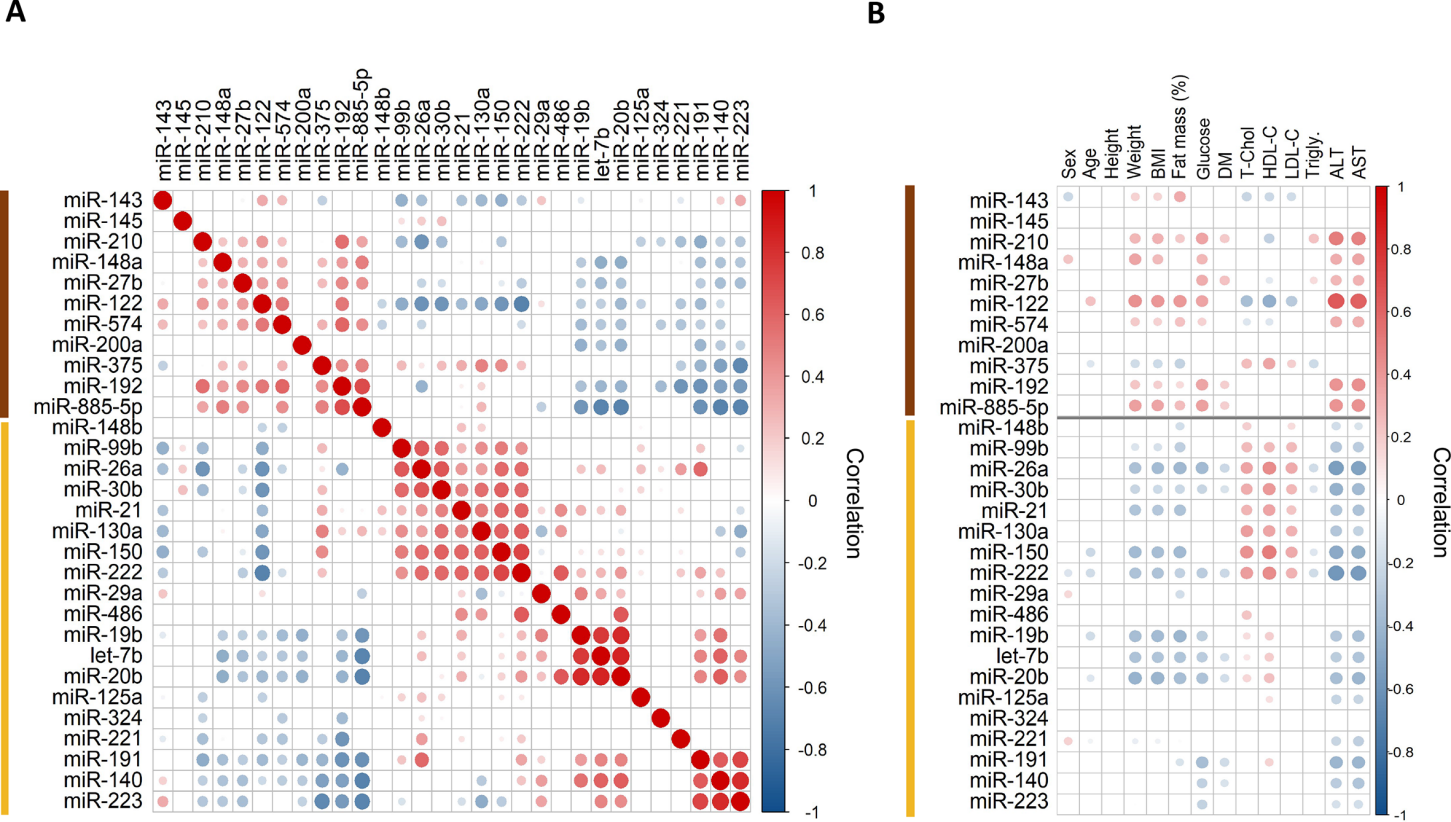
(A) Pairwise Spearman correlation of serum microRNA (miRNA) levels. Hierarchical clustering analysis and heat map matrix illustrating positive and negative co-expression and intracluster and intercluster relationships of pairwise comparisons of miRNAs before bariatric surgery. Only significant correlations are shown (p<0.05, normalized to the global Cq average). The sizes of the circles highlight the strength of correlation. Two distinct clusters emerge: one smaller cluster (brown color) is composed of liver-specific miR-122 and other liver-related miRNAs such as miR-885-5p. The larger cluster (yellow color) contains a wide range of miRNAs from different cellular origins, including the putative adipose tissue-related miRNAs miR-99b, miR-221 and miR-222. (B) Correlations of serum miRNAs to clinical variables. Spearman correlation plot showing only significant correlations before bariatric surgery (p<0.05, normalized to the global Cq average). For the categorical variables sex and diabetes, point-biserial correlation was used. The cluster of liver-related miRNAs (highlighted in brown) is positively correlated (red color) with weight, BMI, percentage fat mass, blood glucose and liver transaminases. The different sizes of the circles highlight the strength of correlation. There was no positive association between BMI and percentage fat mass with serum levels of putative adipose tissue-related miRNAs, miR-99b, miR-221 and miR-222. ALT, alanine aminotransferase; AST, aspartate aminotransferase; BMI, body mass index; DM, type 2 diabetes mellitus; fat mass (%), percentage fat mass; HDL-C, high-density lipoprotein cholesterol; LDL-C, low-density lipoprotein cholesterol; T-Chol, total cholesterol.

Correlation analysis revealed distinct clusters with positive co-expression (figure 2A). The cluster with the most pronounced obesity-induced changes included liver-specific miR-122. Based on previous studies, as well as the human miRNA transcriptomic atlas, this cluster contained other abundant liver miRNAs, that is, miR-192 and miR-885-5p.^34 35^ In a correlation analysis between baseline miRNAs levels with clinical variables in obese patients (figure 2B), miRNAs correlating with miR-122, such as miR-210, miR-192 and miR-885-5p, but not miR-375, which is expressed in islet cells of the pancreas,^36^ were positively correlated with weight, BMI, percentage fat mass and glucose levels, and especially with liver transaminases, further supporting a relationship between these serum miRNAs and liver dysfunction.

### Circulating miRNA changes after bariatric surgery

**Figure 3:**
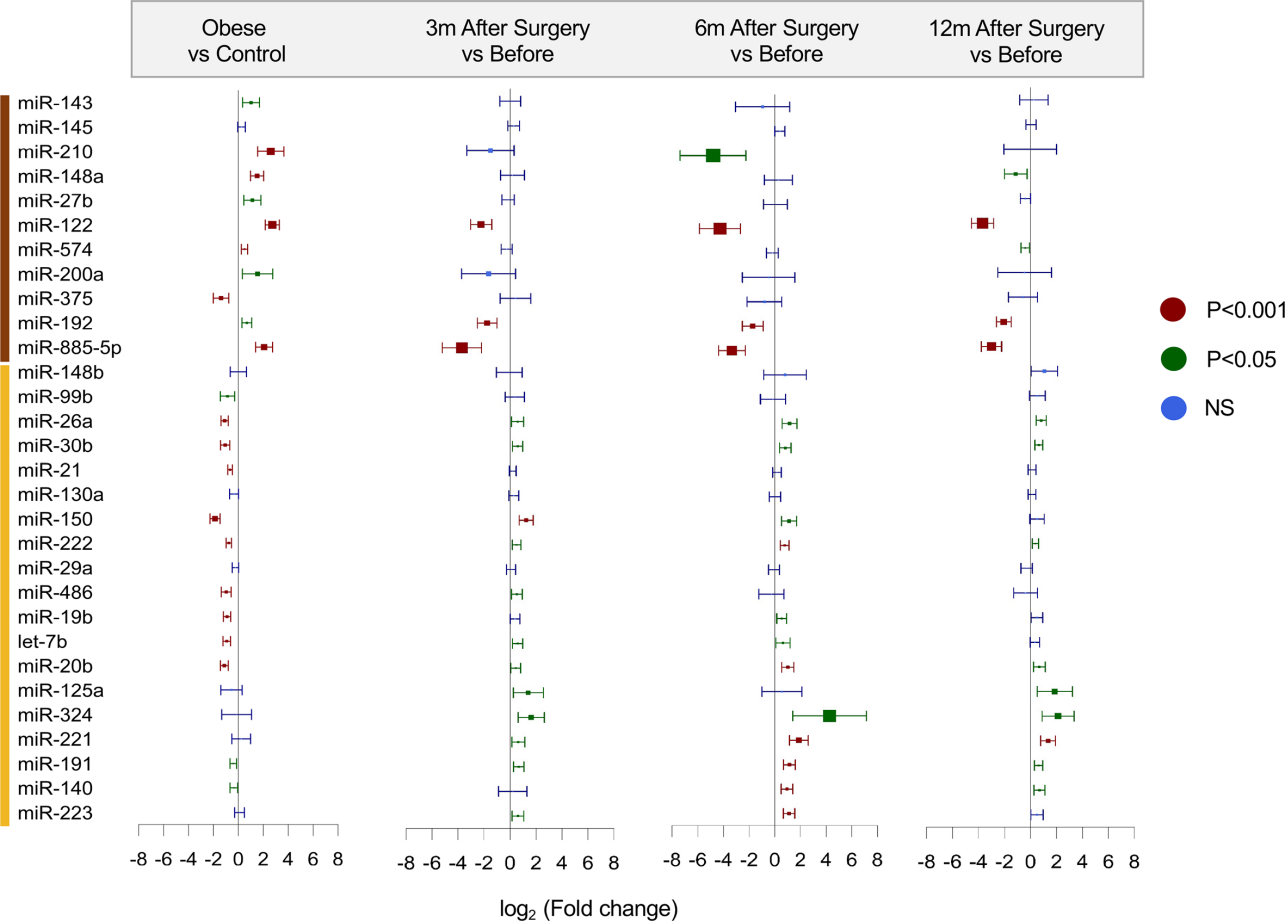
Effect of obesity and bariatric surgery on serum microRNA (miRNA) levels. Forest plots with the corresponding relative abundance ratios (log_2_-fold change, normalized to the global Cq average). Levels of miR-122, miR-885-5p and miR-192 fell within the first 3 months after bariatric surgery. These miRNAs were part of the cluster containing liver-related miRNAs (highlighted in brown color). At 3 months, patients achieved a reduction in body mass index (BMI) of approximately 20%. In contrast, miRNAs within the cluster containing the putative adipose tissue-related miRNAs (yellow color) showed no reduction even at 12 months of follow-up despite a reduction in BMI of almost 40%. The p-values are corrected for multiple testing and the box sizes (shown in blue, green, maroon for the different p-value ranges) are relative to the absolute log_2_-fold change. NS, not significant.

Follow-up samples were available in a subset of patients from the discovery cohort at 3 months (n=29, T=3), 6 months (n=27, T=6) and 12 months (n=27, T=12) after bariatric surgery. Bariatric surgery reduced the elevated serum levels of miR-122, miR-192, miR-885-5p and miR-210 (figure 3; online supplemental tables 3–5). In contrast, many blood cell-derived miRNAs, that is, miR-21,^37^ and putative adipose tissue-derived miRNAs^12 32 33^ were either unchanged, that is, miR-99b, or even increased, that is, miR-221 and miR-222, after bariatric surgery (figure 3; online supplemental table 6). Again, miRNA concentrations that were standardized to the global Cq average (online supplemental table 6) were in agreement with results that used *cel*-miR-39 as an alternative normalization strategy (online supplemental table 7). Given a nearly 40% reduction in the average BMI after 12 months (from 49.6±0.62 before to 30.9±0.91 kg/m^2^ after bariatric surgery), our results suggest that adipose tissue may not be the main source of these circulating miRNAs.

To confirm our key findings, we performed further miRNAs measurements in an independent validation cohort of obese patients with samples before bariatric surgery (n=33) and at follow-up after 12 months (n=14) or 18 months (n=19), respectively. Their clinical characteristics are shown in online supplemental table 8. A comparison of the results from the discovery and the validation cohort is presented in online supplemental table 9, confirming a significant reduction of circulating liver-derived miRNAs after bariatric surgery (online supplemental tables 10 and 11). In contrast, analysis of the putative adipose tissue-derived miRNAs showed no significant changes for miR-99b, miR-222 and miR-221 at either time point despite a pronounced and sustained weight loss over the 18 months of follow-up after bariatric surgery.

### Compartmentalization of circulating miRNAs

**Figure 4:**
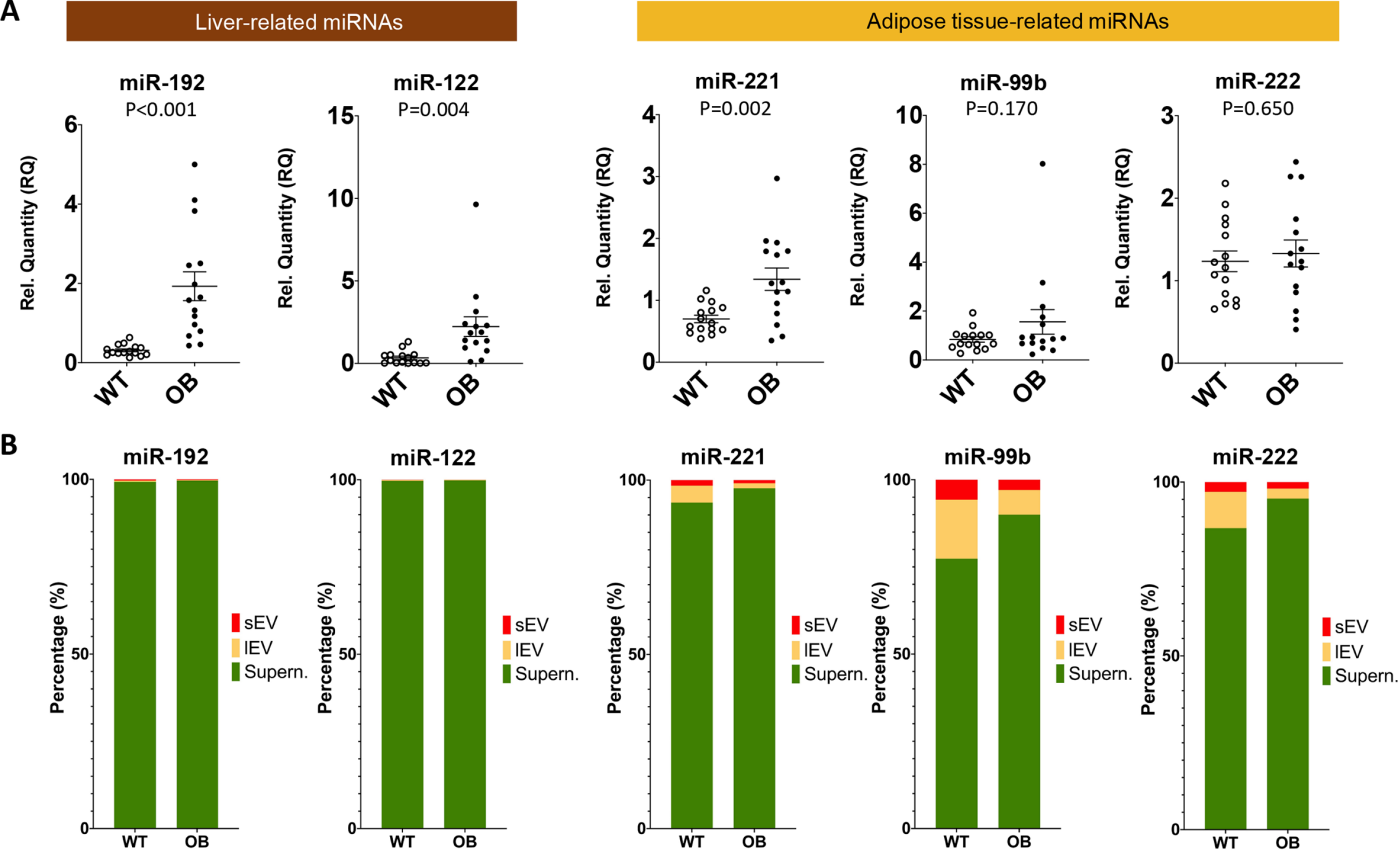
(A) Differences in serum microRNAs (miRNAs) between control C57BL/6 (WT, wild type) and Lep^ob^ mice (OB, obese). (B) Compartmentalization of serum miRNAs. Differential centrifugation was used to separate small extracellular vesicles (sEVs), large extracellular vesicles (lEVs) and EV-depleted supernatant (Supern.) from mouse serum (WT vs Lep^ob^ mice, all male mice aged 14–15 weeks fed on chow diet).

Baseline levels of putative adipose tissue-derived miRNAs were lower in obese patients compared with non-obese controls, that is, miR-99b and miR-222, or were not different, that is, miR-221. Moreover, circulating levels of miR-99b did not change after bariatric surgery despite the substantial weight loss, while miR-221 and miR-222 even increased after bariatric surgery. A possible explanation for these results is that these serum miRNAs are either not adipose tissue-derived or adipose tissue-derived exosomes are not a major contributor to the total circulating miRNA content as suggested previously.^12^ To address the latter question, we harvested serum from 14 to 15 week old wild type and obese-hyperglycemic Lep^ob^ mice and separated EVs by differential centrifugation. The profound increase of liver miRNAs observed in morbidly obese patients could independently be replicated in serum samples of obese-hyperglycemic Lep^ob^ mice (figure 4A). Next, we conducted miRNA analysis in sEVs and lEVs as well as in the EV-depleted supernatant. Liver-derived miR-192-5p and miR-122-5p were predominantly detected in the EV-depleted supernatant. MiR-885-5p was not included as this miRNA is not present in mice. For the putative adipose tissue-derived miRNAs, miR-221, miR-99b and miR-222, their relative miRNA content in EVs was higher than liver-derived miRNAs (figure 4B), but there was no significant change in sEVs isolated from serum of Lep^ob^ mice compared with wild-type controls (online supplemental figure 4). In fact, a reduction of miR-221 and miR-222 was observed in lEVs from Lep^ob^ mice, in opposing direction to the corresponding serum levels. Thus, EVs are a minor contributor to the circulating levels of these putative adipose tissue-derived miRNAs.

## Discussion

The circulating miRNAs that showed the most profound changes in morbidly obese patients formed a distinct co-expression cluster that included liver-specific miR-122, which correlated with body weight, BMI, percentage fat mass and liver transaminase levels. The observed drop of liver miRNAs within the first 3 months after bariatric surgery indicated a metabolic recovery following a timeline correlating with the normalization of transaminase levels. At 3 months, the patients achieved on average a 20% reduction in their BMI. Additional weight loss thereafter had no further effect on circulating levels of liver miRNAs. In contrast, candidate adipose-derived miRNAs, such as miR-99b, which Thomou *et al*^12^ suggested to be a previously undescribed form of adipokine, did not decline after bariatric surgery despite the dramatic weight loss after a 12–18-month follow-up.

Previous work reported higher miR-122, miR-148a and miR-192 circulating levels in obesity.^38^ Altered blood miR-122 and miR-885-5p levels have also been implicated in a variety of liver diseases, including drug-induced liver injury and NAFLD.^39 40^ Prevalence of NAFLD increases in parallel with obesity, metabolic syndrome and diabetes.^41 42^ In patients with NAFLD, miR-122 and miR-192 were among the most upregulated miRNAs in serum.^43^ Elevated miR-122 and miR-192 levels were also part of a plasma signature that predicted postoperative liver dysfunction.^44^ Obese patients showed reduced levels of miR-122 1 year after RYGB while before surgery miR-122 circulating levels correlated with aspartate aminotransferase and alanine aminotransferase.^45^ Other circulating miRNAs that showed profound changes in our study have also been reported to increase with liver damage, that is, miR-210, miR-148a, miR-143, miR-574, miR-27b and miR-200a.^39 40 43 44 46^

Thomou *et al*^12^ suggested a predominant adipose tissue origin of circulating exosomal miRNAs based on the use of adipose tissue-specific Dicer knockout (AdicerKO) mice. Dicer is required for the conversion of most, but not all pre-miRNAs to mature miRNAs.^47^ Liver-related miRNAs with profound changes after bariatric surgery, that is, miR-148a, miR-122, miR-210, were also decreased in exosomes from AdicerKO mice. In the study by Thomou *et al*,^12^ exosomal miRNA profiling in patient serum returned only one miRNA, which was also found in our study to be significantly increased in obesity and reduced after bariatric surgery: miR-210 was upregulated in patients with congenital generalized lipodystrophy but not dysregulated in HIV-associated lipodystrophy (n=4 each and n=4 controls). To the best of our knowledge, the putative adipose tissue-derived miRNAs have not been measured in morbidly obese patients so far. Despite the fact that we detected high levels of adipose tissue-derived miRNAs in healthy human subcutaneous adipose tissue, the present data in morbidly obese patients refute that serum miRNAs are predominantly adipose tissue derived or that white adipose tissue-derived exosomes are a major contributor to the total circulating levels of miRNAs.

One potential explanation for the discrepancy between our findings in morbidly obese patients and the ones reported by Thomou *et al*^12^ in AdicerKO mice is that brown adipose tissue is prominent in mice. Rodents and other small mammals have copious brown fat, whereas humans have very little after childhood.^48^ Thus, while liver miRNAs remain conserved across species, the composition of adipose-derived miRNAs, would be different between mice and humans. Given the analytical challenges and the lack of scalable methods to obtain pure vesicular preparations, we did not attempt to isolate EVs from human serum. However, the putative adipose tissue-derived miRNAs miR-99b, miR-221 and miR-222 showed no increase in the vesicle fractions of hyperglycemic Lep^ob^ mice. It should be noted that, although plasma concentrations of putative adipose-tissue derived miRNAs did not change with weight loss, changes in adipose tissue function could still affect circulating miRNAs. Dynamic changes in the production or removal of circulating miRNAs might not be reflected in static concentrations.

In summary, morbid obesity was associated with a profound increase in liver-derived miRNAs. The elevated serum levels of liver-derived miRNAs dropped within 3 months after bariatric surgery. This was associated with a recovery of parameters related to liver dysfunction and reversal of type 2 diabetes, but not to further weight loss, suggesting that the main cause is a metabolic improvement rather than the loss of white adipose tissue.
