# Supplementary material for: Metabolic recovery after weight loss surgery is reflected in serum microRNAs
Source: BMJ Open Diabetes Res Care. 2020 Oct 28;8(2):e001441. doi: 10.1136/bmjdrc-2020-001441 (PMC7594349; doi:10.1136/bmjdrc-2020-001441)
Supplement: Supplementary data [file bmjdrc-2020-001441supp001.pdf]

Online Supplemental Data

Metabolic recovery after weight loss surgery is reflected in serum microRNAs

Susana Sangiao-Alvarellos [1,2,3 *], Konstantinos Theofilatos [1], Temo Barwari [1], Clemens Gutmann[1], Kaloyan Takov[1], Bhawana Singh[1], Paula Juiz-Valiña [2,3], Bárbara María Varela-Rodríguez [2,3], Elena Outeiriño-Blanco [4], Elisa Duregotti [1], Anna Zampetaki [1], Lukas Lunger [5], Christoph Ebenbichler [5], Herbert Tilg [5], María Jesús García-Brao [6], Peter Willeit [7,8], Enrique Mena [6], Stefan Kiechl [8,9], Fernando Cordido [2,3,4], Manuel Mayr [1 *].

1. King's British Heart Foundation Centre, King's College London, London, United Kingdom

2. Endocrine, nutritional and metabolic diseases group. Department of Physiotherapy, Medicine and Biomedical Sciences. Faculty of Health Sciences. University of A Coruña, A Coruña, Spain.

3. Instituto de Investigación Biomédica de A Coruña (INIBIC), A Coruñ, Spain.

4. Department of Endocrinology, University Hospital A Coruña, A Coruña, Spain.

5. Department of Internal Medicine I, Gastroenterology, Endocrinology & Metabolism, Medical University of Innsbruck, Innsbruck, Austria.

6. Department of Digestive and General Surgery, University Hospital A Coruña, A Coruña, Spain.

7. Department of Public Health and Primary Care, University of Cambridge, Cambridge, United Kingdom

8. Department of Neurology, Medical University of Innsbruck, Innsbruck, Austria.

9. VASCage, Research Centre on Vascular Ageing and Stroke, Innsbruck, Austria

Supplemental Figure 1. Dot blot characterization of EV fractions from mouse plasma.

Levels of syntenin-1 (SDCBP, small extracellular vesicle marker) and apolipoprotein-B (APOB, marker of lipoprotein contamination) in fractionated plasma from wild-type (WT) and Lep^{ob} (OB) mice.

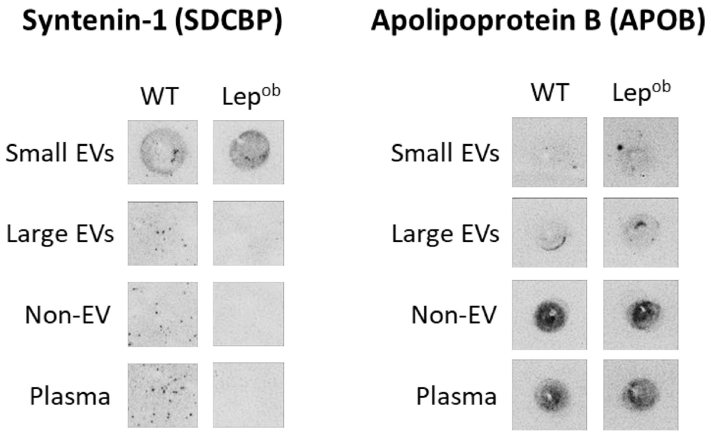

Supplemental Figure 2. Changes in serum miRNAs between control and obese patients normalized to *cel*-miR-39.

Volcano plot for miRNA levels normalized to an exogenous miRNA, *cel*-miR-39, spiked in during RNA extraction. Statistical comparison was conducted using the e-bayes method of the limma package. P-values were corrected for multiple testing and are shown in blue, green, maroon for the different p-values ranges. Liver-specific miR-122 and liver-related miR-885-5p were amongst the miRNAs with the most elevated serum levels in obese patients (n=155) compared to controls (n=47).

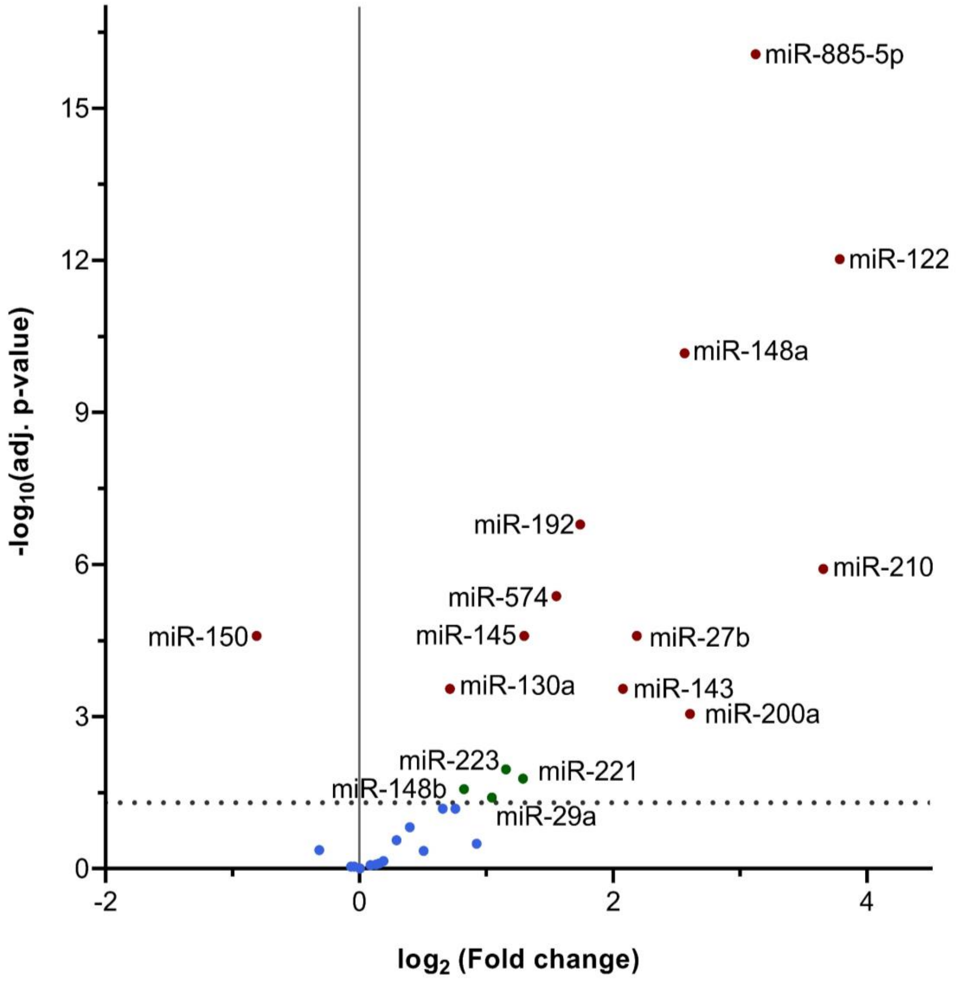

Supplemental Figure 3. Bean plots for miRNAs normalized to *cel*-miR-39.

Again using exogenous *cel*-miR-39 for normalization, serum levels of miR-122, miR-885-5p and miR-192 were markedly elevated with obesity, while serum levels of putative adipose-tissue related miRNAs (miR-99b, miR-222) were not significantly different between control and obese patients.

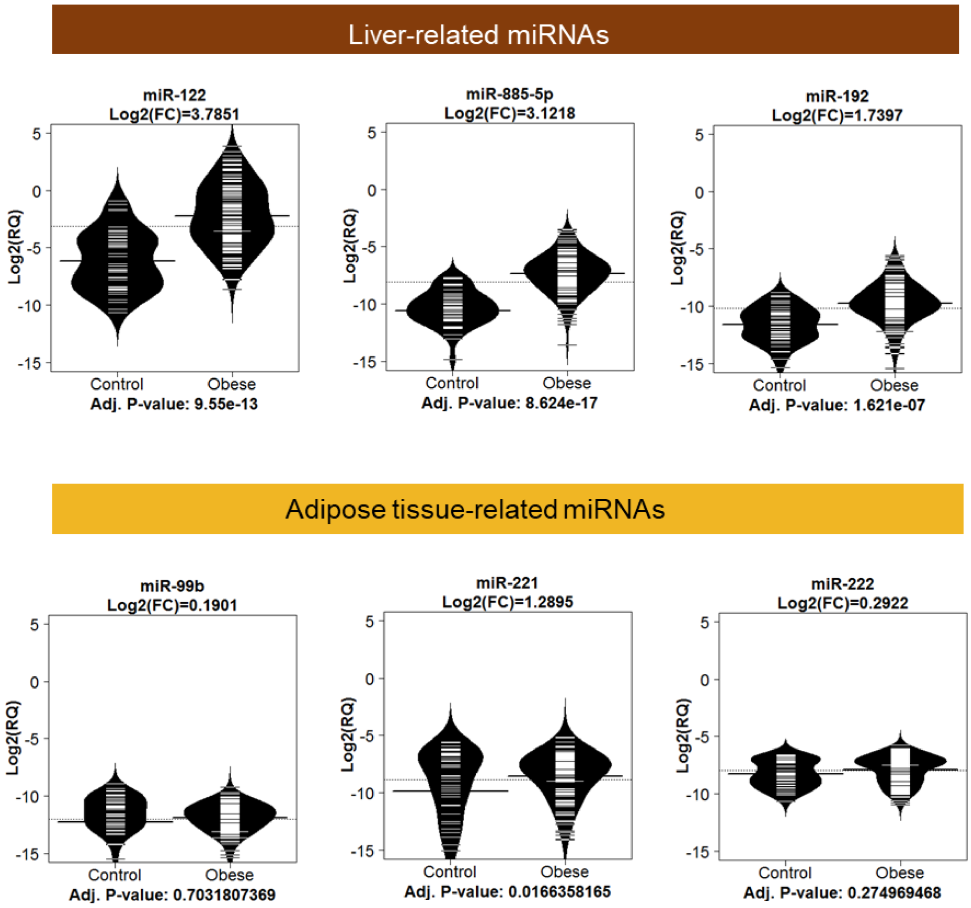

Supplemental Figure 4. miRNA compartmentalization.

Bar graphs showing miRNA differences (relative quantity, RQ) between wildtype (WT) and Lep^{ob} (OB) mice. Small extracellular vesicles (sEVs), large EVs (lEVs) and EV-depleted supernatant were obtained from 100 ul of serum (n=13 mice per group). The increase in liver-derived serum miR-122 and miR-192 was reflected in the EV-depleted supernatant with corresponding trends being observed in both EV fractions. Note that the different scale of the y-axis reflects the higher miRNA content in the EV-depleted supernatant compared to the sEV and lEV fraction. The putative adipose tissue-derived miRNAs miR-99b, miR-221 and miR-222 showed no increase in the EV fractions of Lep^{ob} mice. p-values were derived from Student's t-tests.

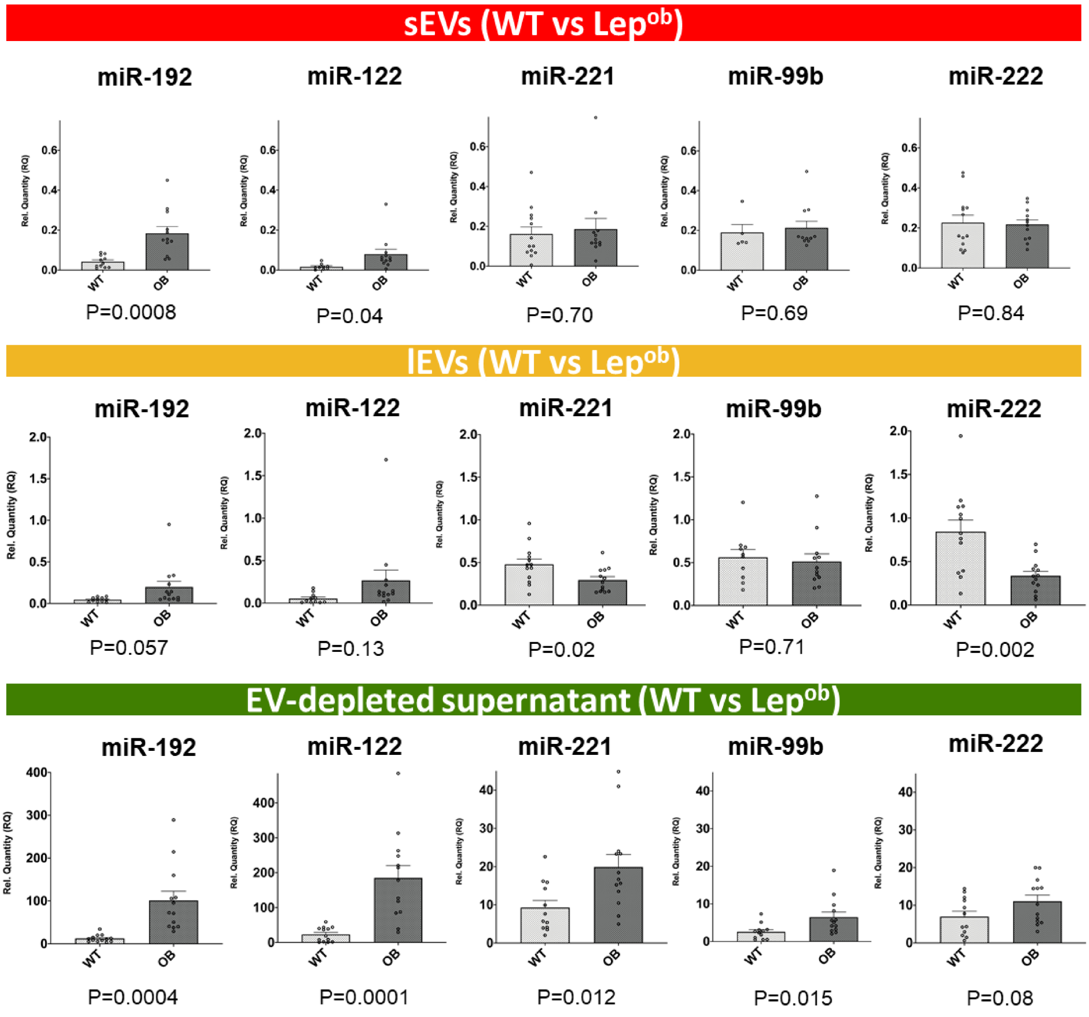

Supplemental Table 1. Anthropometric and biochemical parameters of the discovery cohort.

Values are expressed as mean ± SD. Serial adipose tissue samples were obtained from obese patients before and at various intervals after bariatric surgery i.e. 3, 6 and 12 months after surgery. T=0 indicates timepoint before surgery, T=3 indicates timepoint 3 months after surgery, T=6 indicates timepoint 6 months after surgery and T=12 indicates timepoint 12 months after surgery. The follow-up measurement post-surgery was obtained at different intervals from different patients. Mann Whitney U-test and Fisher Exact test were used for the control vs obese comparison of the numerical and categorical clinical characteristics of the cohort respectively. Wilcoxon Signed rank test (paired analysis) and Fisher Exact test were used for the before and after surgery comparisons of the numerical and categorical clinical characteristics of the cohort, respectively.

	Control	Obese, Before Surgery (T=0)	Control Vs Obese P-value	Obese, 3 Months After Surgery (T=3)	Obese, 6 Months After Surgery (T=6)	Obesity, 12 Months After Surgery (T=12)	Before and after surgery (3, 6, 12 months respectively) P-value
**Sample Size**	47	155		29	27	28	
**Sex**	20♂, 27♀	47♂, 108♀	0.156	10♂, 19♀	10♂, 17♀	7♂, 21♀	
**Age (years)**	39.7 ± 10.90	45.3 ± 9.21	<0.001				
**Body Weight (Kg)**	69.46 ± 13.88	135.01 ± 25.91	<0.001	107.97 ± 25.07	98.72 ± 20.98	82.75 ± 14.76	(<0.001, <0.001, <0.001)
**BMI (kg/m^2)**	24.3 ± 3.49	49.2 ± 8.54	<0.001	39.4 ± 7.53	35.9 ± 6.10	30.6 ± 4.8	(<0.001, <0.001, <0.001)
**Fat mass (%)**	24.7 ± 8.43	48.48 ± 6.22	<0.001	41.15 ± 8.40	35.35 ± 6.60	31.44 ± 8.63	(<0.001, <0.001, <0.001)
**DM2 (%)**	0%	36.10%	<0.001	3.44%	7.41%	3.57%	(<0.001, <0.001, <0.001)
**Anti-diabetic drugs (%)**	0%	35.48%	<0.001	3.44%	7.41%	3.57%	(<0.001, <0.001, <0.001)
**Anti-lipid drugs (%)**	0%	34.84%	<0.001	13.79%	7.41%	7.14%	(<0.001, <0.001, <0.001)
**T-Chol (mg/dL)**	194.4 ± 35.51	145 ± 38.47	<0.001	179.57 ± 33.12	176.32 ± 36.43	179.44 ± 34.24	(<0.001, 0.325, 0.070)
**HDL-C (mg/dL)**	59.9 ± 14.05	32.02 ± 5.35	<0.001	39.29 ± 10.99	45.17 ± 8.94	58.64 ± 19.79	(<0.001, 0.003, <0.001)
**LDL-C (mg/dL)**	119.7 ± 32.08	87.82 ± 29.76	<0.001	120.81 ± 33.71	114.53 ± 34.92	109.85 ± 33.02	(<0.001, 0.118, 0.182)
**Triglycerides (mg/dL)**	81.79 ± 30.51	118.39 ± 57.27	<0.001	123.11 ± 50.30	92.56 ± 31.44	81.48 ± 24.98	(0.885, <0.001, <0.001)
**ALT (UI/L)**	19.18 ± 4.46	41.64 ± 27.89	<0.001	23.03 ± 9.64	19.64 ± 9.92	19.52 ± 7.14	(<0.001, 0.022, 0.002)
**AST (UI/L)**	20.06 ± 10.56	51.08 ± 33.86	<0.001	26.96 ± 13.52	22.21 ± 42.61	24.46 ± 16.40	(<0.001, <0.001, 0.003)
**IGF-1 (ng/mL)**	156 ± 57.18	89.69 ± 58.89	<0.001	94.27 ± 34.25	111.85 ± 42.66	113.52 ± 28.89	(0.256, 0.0.564, 0.001)
**Insulin (µUI/L)**	5.12 ± 3.56	8.31 ± 7.47	<0.001	8.91 ± 4.20	8.67 ± 4.73	6.74 ± 2.38	(0.239, 0.0025, <0.001)

**Fasting glucose (mg/dL)**	89.48 ± 8.23	108.72 ± 31.50	<0.001	95.55 ± 19.98	90.55 ± 14.91	89.22 ± 23.97	(0.023, <0.001, <0.001)
**GH (ng/mL)**	1.59 ± 2.54	1.21 ± 2.37	0.647	1.44 ± 1.40	2.44 ± 3.22	3.03 ± 2.70	(0.940, 0.007, 0.325)
**C-Peptide (ng/mL)**	1.71 ± 1.30	2.48 ± 1.62	<0.001	2.35 ± 0.59	2.24 ± 3.22	1.93 ± 0.37	(0.496, 0.005, <0.001)
**ApoA (mg/dL)**	168.80 ± 32.56	110.4 ± 26.52	<0.001	135.48 ± 44.75	158.71 ± 33.62	162.49 ± 33.71	(0.071, 0.034, <0.001)
**ApoB (mg/dL)**	103.2 ± 31.19	86.5 ± 32.12	0.011	125.92 ± 68.88	124.51 ± 55.70	92.79 ± 32.12	(<0.001, 0.052, 0.683)
**CRP (mg/dL)**	0.18 ± 0.34	0.99 ± 1.12	<0.001	0.66 ± 0.75	0.81 ± 0.99	0.28 ± 0.48	(0.025, 0.981, <0.001)
**HbA1c (%)**	5.15 ± 0.48	5.45 ± 1.49	0.865	5.7 ± 0.86	5.68 ± 0.68	5.94 ± 1.32	(0.021, 0.027, 0.163)
**HOMA-IR**	1.26 ± 1.17	2.33 ± 2.37	<0.001	2.04 ± 0.97	1.98 ± 1.25	1.50 ± 0.58	(0.045, <0.001, <0.001)
**QUICKI**	0.39 ± 0.04	0.36 ± 0.05	<0.001	0.36 ± 0.04	0.36 ± 0.04	0.37 ± 0.04	(0.191, <0.001, 0.773)
**HICS**	0.38 ± 0.22	0.40± 0.19	0.366	0.39 ± 0.23	0.31 ± 0.11	0.33 ± 0.14	(0.405, 0.075, 0.203)

BMI: body mass index, DM2: diabetes mellitus 2; T-Chol: total cholesterol, HDL-C: high-density lipoprotein cholesterol, LDL-C: low-density lipoprotein cholesterol, ALT: alanine aminotransferase, AST: aspartate aminotransferase, IGF-1: Insulin-like growth factor 1, GH: growth hormone, ApoA: Apolipoprotein A, ApoB: Apolipoprotein B, CRP: C-reactive protein, HbA1c: Glycated haemoglobin, HOMA-IR: homeostatic model assessment for insulin resistance, QUICKI: Quantitative insulin sensitivity check index, HICS: Hepatic Insulin Clearance Score calculated as the ratio of C-peptide to insulin.

Supplemental Table 2: Obese vs control comparison in the discovery cohort normalized to cel-miR-39.

Levels of miRNA were calculated in obese versus control participants (unpaired analysis) using the e-bayes algorithm of the limma package and correcting for age, sex and statin uses. Adjustment of p-values for multiple testing was conducted with Benjamini-Hochberg algorithm. In the table below, FC denotes fold-change; CIs denote limits of confidence interval for FC; AveExpr denotes average expression; t denotes moderated t-statistic; P.Value denotes raw p-value; adj.P.Value denotes adjusted p-value or q-value and B denotes log-odds that the miRNA is differentially expressed.

	FC	Low CI	High CI	AveExpr	t	P.Value	adj.P.Val	B
miR-885-5p	8.70	5.59	13.55	0.00	9.64	0.00	0.00	30.84
miR-122	13.79	7.27	26.15	0.11	8.08	0.00	0.00	20.99
miR-148a	5.91	3.66	9.54	0.00	7.30	0.00	0.00	16.41
miR-192	3.34	2.22	5.02	0.00	5.84	0.00	0.00	8.54
miR-210	12.59	4.98	31.84	0.00	5.39	0.00	0.00	6.37
miR-574	2.93	1.93	4.45	0.00	5.09	0.00	0.00	4.99
miR-27b	4.55	2.38	8.68	0.00	4.62	0.00	0.00	2.98
miR-145	2.46	1.68	3.61	0.00	4.62	0.00	0.00	2.98
miR-150	0.57	0.45	0.73	0.01	-4.60	0.00	0.00	2.88
miR-143	4.22	2.06	8.62	0.00	3.97	0.00	0.00	0.41
miR-130a	1.64	1.28	2.10	0.00	3.96	0.00	0.00	0.39
miR-200a	6.08	2.28	16.19	0.00	3.64	0.00	0.00	-0.76
miR-223	2.22	1.28	3.86	0.50	2.86	0.00	0.01	-3.15
miR-221	2.44	1.27	4.71	0.00	2.69	0.01	0.02	-3.60
miR-29a	1.77	1.13	2.78	0.00	2.49	0.01	0.03	-4.09
miR-148b	2.06	1.12	3.81	0.00	2.33	0.02	0.04	-4.48
miR-140	1.69	1.03	2.76	0.00	2.10	0.04	0.07	-4.97
miR-191	1.58	1.02	2.43	0.01	2.07	0.04	0.07	-5.02
miR-21	1.32	0.95	1.82	0.02	1.67	0.10	0.15	-5.76
miR-222	1.22	0.91	1.65	0.00	1.34	0.18	0.27	-6.26
miR-324	1.90	0.67	5.34	0.00	1.22	0.22	0.32	-6.41
miR-375	0.80	0.52	1.23	0.00	-1.01	0.32	0.43	-6.64
miR-125a	1.42	0.69	2.93	0.00	0.96	0.34	0.44	-6.69
miR-99b	1.14	0.73	1.79	0.00	0.58	0.56	0.70	-6.98
miR-19b	1.11	0.69	1.79	0.11	0.44	0.66	0.79	-7.05
let-7b	1.10	0.67	1.79	0.01	0.37	0.71	0.82	-7.08
miR-486	1.06	0.72	1.57	0.02	0.30	0.77	0.85	-7.10
miR-20b	0.96	0.59	1.55	0.00	-0.19	0.85	0.91	-7.13
miR-26a	0.97	0.68	1.40	0.00	-0.16	0.88	0.91	-7.13
miR-30b	1.00	0.75	1.35	0.00	0.02	0.98	0.98	-7.15

Supplemental Table 3: After surgery (3 months) vs Before surgery comparison in the discovery cohort normalized to cel-miR-39.

Detailed results of the statistical comparison in the discovery cohort for miRNA levels 3 months after surgery and before bariatric surgery (paired analysis) using the e-bayes algorithm of the limma package and paired analysis correcting for statin use. Adjustment of p-values for multiple testing was conducted with the Benjamini-Hochberg algorithm. In the table below, FC denotes fold-change; CIs denote limits of confidence interval for FC; AveExpr denotes average expression; t denotes moderated t-statistic; P.Value denotes raw p-value; adj.P.Value denotes adjusted p-value or q-value and B denotes log-odds that the miRNA is differentially expressed.

	FC	Low CI	High CI	AveExpr	t	P.Value	adj.P.Val	B
miR-122	0.18	0.09	0.37	0.03	4.99	0.00	0.00	2.44
miR-192	0.25	0.14	0.46	0.00	4.70	0.00	0.00	1.68
miR-210	0.08	0.02	0.43	0.00	3.08	0.00	0.05	-2.44
miR-150	2.01	1.23	3.30	0.01	-2.89	0.01	0.06	-2.88
miR-125a	0.08	0.01	0.66	0.00	2.43	0.02	0.11	-3.85
miR-885-5p	0.25	0.08	0.81	0.00	2.41	0.02	0.11	-3.90
miR-140	1.92	1.03	3.58	0.00	-2.13	0.04	0.18	-4.45
miR-574	0.71	0.45	1.12	0.00	1.53	0.14	0.51	-5.44
miR-27b	0.76	0.50	1.17	0.00	1.29	0.21	0.54	-5.76
miR-30b	1.27	0.86	1.87	0.00	-1.27	0.21	0.54	-5.79
miR-375	0.56	0.22	1.46	0.00	1.24	0.23	0.54	-5.83
miR-191	1.35	0.80	2.29	0.01	-1.17	0.25	0.54	-5.91
miR-222	1.20	0.87	1.67	0.00	-1.16	0.26	0.54	-5.92
miR-324	2.52	0.46	13.89	0.00	-1.11	0.28	0.54	-5.97
miR-26a	1.27	0.81	1.98	0.00	-1.09	0.28	0.54	-5.99
miR-221	1.32	0.78	2.26	0.00	-1.08	0.29	0.54	-6.00
miR-486	1.22	0.80	1.85	0.01	-0.97	0.34	0.60	-6.11
let-7b	1.27	0.75	2.13	0.01	-0.93	0.36	0.60	-6.14
miR-223	1.29	0.68	2.45	0.34	-0.81	0.42	0.67	-6.25
miR-148b	1.58	0.44	5.70	0.00	-0.73	0.47	0.70	-6.31
miR-20b	1.16	0.69	1.94	0.00	-0.57	0.57	0.77	-6.42
miR-29a	0.89	0.59	1.36	0.00	0.55	0.59	0.77	-6.43
miR-145	1.12	0.73	1.72	0.00	-0.55	0.59	0.77	-6.43
miR-143	0.86	0.44	1.68	0.00	0.46	0.65	0.81	-6.48
miR-19b	1.11	0.65	1.89	0.07	-0.39	0.70	0.84	-6.50
miR-99b	1.12	0.53	2.35	0.00	-0.30	0.77	0.88	-6.54
miR-130a	1.04	0.64	1.69	0.00	-0.16	0.87	0.96	-6.57
miR-21	0.98	0.63	1.52	0.01	0.09	0.93	0.96	-6.58
miR-148a	0.97	0.48	1.98	0.00	0.09	0.93	0.96	-6.58
miR-200a	1.07	0.09	12.73	0.00	-0.05	0.96	0.96	-6.58

Supplemental Table 4: to After surgery (6 months) vs Before surgery comparison in the discovery cohort normalized to cel-miR-39.

Detailed results of the statistical comparison in the discovery cohort for miRNA expression levels 6 months after surgery and before bariatric surgery (paired analysis) using the e-bayes algorithm of the limma package and paired analysis correcting for statin use. Adjustment of p-values for multiple testing was conducted with the Benjamini-Hochberg algorithm. In the table below, FC denotes fold-change; CIs denote limits of confidence interval for FC; AveExpr denotes average expression; t denotes moderated t-statistic; P.Value denotes raw p-value; adj.P.Value denotes adjusted p-value or q-value and B denotes log-odds that the miRNA is differentially expressed.

	FC	Low CI	High CI	AveExpr	T	P.Value	adj.P.Val	B
miR-885-5p	0.08	0.04	0.16	0.00	7.60	0.00	0.00	8.09
miR-192	0.25	0.14	0.45	0.00	4.85	0.00	0.00	1.50
miR-122	0.09	0.03	0.26	0.01	4.84	0.00	0.00	1.48
miR-150	1.78	1.27	2.50	0.00	-3.51	0.00	0.01	-1.78
miR-221	3.01	1.49	6.06	0.00	-3.25	0.00	0.02	-2.40
miR-26a	1.82	1.14	2.92	0.00	-2.64	0.01	0.07	-3.75
miR-191	1.80	1.10	2.94	0.00	-2.47	0.02	0.09	-4.10
miR-324	0.03	0.00	0.64	0.00	2.36	0.03	0.10	-4.32
miR-210	16.54	1.34	204.54	0.01	-2.30	0.03	0.10	-4.43
miR-223	1.77	1.00	3.15	0.15	-2.06	0.05	0.15	-4.88
miR-30b	1.45	0.98	2.13	0.00	-1.96	0.06	0.17	-5.06
miR-20b	1.66	0.92	2.99	0.00	-1.79	0.09	0.21	-5.35
miR-375	0.47	0.20	1.13	0.00	1.77	0.09	0.21	-5.37
miR-222	1.41	0.93	2.12	0.00	-1.72	0.10	0.21	-5.46
miR-574	0.72	0.44	1.17	0.00	1.41	0.17	0.35	-5.92
miR-140	1.46	0.81	2.60	0.00	-1.34	0.19	0.36	-6.01
miR-99b	1.64	0.59	4.55	0.00	-1.01	0.32	0.49	-6.38
miR-486	0.68	0.31	1.50	0.01	1.00	0.33	0.49	-6.39
miR-29a	0.79	0.49	1.29	0.00	1.00	0.33	0.49	-6.39
let-7b	1.28	0.77	2.14	0.00	-1.00	0.33	0.49	-6.39
miR-130a	0.83	0.54	1.29	0.00	0.85	0.40	0.57	-6.53
miR-143	0.42	0.05	3.80	0.00	0.82	0.42	0.58	-6.56
miR-19b	1.19	0.72	1.98	0.03	-0.73	0.47	0.62	-6.63
miR-27b	0.85	0.39	1.84	0.00	0.43	0.67	0.78	-6.80
miR-200a	0.59	0.04	7.81	0.01	0.42	0.68	0.78	-6.80
miR-21	0.92	0.61	1.39	0.01	0.40	0.69	0.78	-6.81
miR-148b	1.45	0.18	11.71	0.00	-0.37	0.72	0.78	-6.83
miR-145	1.08	0.68	1.72	0.00	-0.36	0.72	0.78	-6.83
miR-125a	1.19	0.06	23.30	0.00	-0.12	0.91	0.94	-6.89
miR-148a	1.00	0.45	2.21	0.00	0.00	1.00	1.00	-6.90

Supplemental Table 5: After surgery (12 months) vs Before surgery comparison in the discovery cohort normalized to cel-miR-39.

Detailed results of the statistical comparison in the discovery cohort for miRNA levels 12 months after surgery and before bariatric surgery (paired analysis), using the e-bayes algorithm of the limma package and paired analysis correcting for statin use. Adjustment of p-values for multiple testing was conducted with the Benjamini-Hochberg algorithm. In the table below, FC denotes fold-change; CIs denote limits of confidence interval for FC; AveExpr denotes average expression; t denotes moderated t-statistic; P.Value denotes raw p-value; adj.P.Value denotes adjusted p-value or q-value and B denotes log-odds that the miRNA is differentially expressed.

	FC	Low CI	High CI	AveExpr	T	P.Value	adj.P.Val	B
**miR-122**	0.09	0.05	0.15	0.02	9.38	0.00	0.00	12.28
**miR-885-5p**	0.14	0.09	0.23	0.00	8.46	0.00	0.00	10.30
**miR-192**	0.27	0.18	0.43	0.00	6.04	0.00	0.00	4.51
**miR-30b**	1.78	1.29	2.45	0.00	-3.71	0.00	0.01	-1.41
**miR-26a**	2.01	1.36	2.97	0.00	-3.68	0.00	0.01	-1.47
**miR-221**	2.90	1.58	5.33	0.00	-3.61	0.00	0.01	-1.64
**miR-150**	1.62	1.20	2.19	0.00	-3.30	0.00	0.01	-2.39
**miR-210**	0.07	0.01	0.48	0.00	2.87	0.01	0.03	-3.39
**miR-148a**	0.52	0.32	0.86	0.00	2.70	0.01	0.04	-3.77
**miR-191**	1.76	1.11	2.80	0.01	-2.50	0.02	0.06	-4.18
**miR-99b**	1.63	1.07	2.50	0.00	-2.36	0.03	0.07	-4.46
**miR-140**	1.84	1.07	3.16	0.00	-2.32	0.03	0.07	-4.54
**miR-222**	1.48	1.03	2.13	0.00	-2.21	0.04	0.08	-4.76
**miR-20b**	1.83	1.04	3.23	0.00	-2.20	0.04	0.08	-4.79
**miR-223**	1.62	0.95	2.74	0.29	-1.88	0.07	0.14	-5.36
**miR-19b**	1.61	0.95	2.73	0.05	-1.84	0.08	0.15	-5.42
**let-7b**	1.44	0.89	2.35	0.00	-1.55	0.13	0.24	-5.88
**miR-324**	0.33	0.06	1.70	0.00	1.39	0.18	0.29	-6.10
**miR-21**	1.24	0.86	1.77	0.01	-1.21	0.24	0.37	-6.32
**miR-130a**	1.23	0.85	1.78	0.00	-1.17	0.25	0.38	-6.38
**miR-375**	1.52	0.67	3.46	0.00	-1.04	0.31	0.44	-6.52
**miR-125a**	0.54	0.09	3.27	0.00	0.71	0.49	0.63	-6.81
**miR-145**	1.17	0.74	1.87	0.00	-0.70	0.49	0.63	-6.81
**miR-574**	0.86	0.55	1.35	0.00	0.68	0.50	0.63	-6.82
**miR-27b**	0.87	0.53	1.41	0.00	0.61	0.55	0.66	-6.87
**miR-148b**	0.72	0.16	3.13	0.00	0.46	0.65	0.72	-6.95
**miR-143**	0.70	0.14	3.54	0.00	0.46	0.65	0.72	-6.95
**miR-486**	0.88	0.43	1.79	0.01	0.38	0.71	0.74	-6.99
**miR-200a**	1.59	0.10	26.24	0.00	-0.34	0.73	0.74	-7.00
**miR-29a**	0.93	0.58	1.49	0.00	0.33	0.74	0.74	-7.01

Supplemental Table 6: miRNA changes in the discovery cohort normalized to the global Ct average.

Tables showing miRNA comparisons in obese patients after and before bariatric surgery. Using global Ct average normalization in the discovery cohort, a statistically significant reduction of three circulating liver-derived miRNAs was observed (brown color): miR-122, miR-885-5p and miR-192. The elevated serum levels of these liver-related miRNAs dropped after bariatric surgery. The putative adipose tissue-related miRNAs (yellow color) miR-99b, miR-221 and miR-222 do not show a significant reduction after weight loss surgery. FC denotes fold-change; CIs denote limits of confidence interval for FC; Adj.p-value denotes adjusted p-value or q-value.

	Discovery Cohort - Obese vs Control			
	FC	Low CI	High CI	Adj.p-value
miR-122	6.62	4.47	9.79	1.98E-16
miR-885-5p	4.19	2.61	6.71	3.46E-08
miR-192	1.60	1.22	2.11	0.001
miR-99b	0.55	0.37	0.82	0.005
miR-221	1.17	0.70	1.97	0.609
miR-222	0.59	0.51	0.68	1.71E-10

	Discovery Cohort - 3 Months After Surgery vs Before			
	FC	Low CI	High CI	Adj.p-value
miR-122	0.21	0.12	0.38	5.53E-06
miR-885-5p	0.08	0.03	0.22	2.47E-05
miR-192	0.30	0.18	0.50	4.50E-05
miR-99b	1.29	0.78	2.15	0.407
miR-221	1.56	1.10	2.21	0.038
miR-222	1.42	1.13	1.78	0.016

	Discovery Cohort - 6 Months After Surgery vs Before			
	FC	Low CI	High CI	Adj.p-value
miR-122	0.05	0.02	0.16	0.000
miR-885-5p	0.10	0.05	0.20	2.36E-05
miR-192	0.30	0.17	0.53	0.001
miR-99b	0.91	0.46	1.79	0.843
miR-221	3.67	2.21	6.09	0.000
miR-222	1.71	1.36	2.16	0.000

	Discovery Cohort - 12 Months After Surgery vs Before			
	FC	Low CI	High CI	Adj.p-value
miR-122	0.08	0.04	0.14	1.05E-07
miR-885-5p	0.12	0.07	0.21	3.73E-07
miR-192	0.24	0.16	0.36	8.51E-07
miR-99b	1.43	0.94	2.19	0.133
miR-221	2.55	1.72	3.77	0.000
miR-222	1.30	1.09	1.55	0.013

Supplemental Table 7: miRNA changes in the discovery cohort normalized to cel-miR-39.

Tables showing miRNA comparisons in obese patients after and before bariatric surgery. Using an exogenous miRNA for normalization in the discovery cohort, a statistically significant reduction of three circulating liver-derived miRNAs was observed (brown color): miR-122, miR-885-5p and miR-192. The elevated serum levels of these liver-related miRNAs dropped after bariatric surgery. The putative adipose tissue-related miRNAs (yellow color) miR-99b, miR-221 and miR-222 do not show a significant reduction after weight loss surgery. FC denotes fold-change; CIs denote limits of confidence interval for FC; Adj.p-value denotes adjusted p-value or q-value.

	Discovery Cohort – Obese vs Control			
	FC	Low CI	High CI	Adj.p-value
miR-122	13.79	7.27	26.15	9.55E-13
miR-885-5p	8.70	5.59	13.55	8.62E-17
miR-192	3.34	2.22	5.02	1.62E-07
miR-99b	1.14	0.73	1.79	0.703
miR-221	2.44	1.27	4.71	0.017
miR-222	1.22	0.91	1.65	0.275

	Discovery Cohort - 3 Months After Surgery vs Before			
	FC	Low CI	High CI	Adj.p-value
miR-122	0.18	0.09	0.37	0.001
miR-885-5p	0.25	0.08	0.81	0.112
miR-192	0.25	0.14	0.46	0.001
miR-99b	1.12	0.53	2.35	0.883
miR-221	1.32	0.78	2.26	0.543
miR-222	1.20	0.87	1.67	0.543

	Discovery Cohort - 6 Months After Surgery vs Before			
	FC	Low CI	High CI	Adj.p-value
miR-122	0.09	0.03	0.26	0.001
miR-885-5p	0.08	0.04	0.16	2.43E-06
miR-192	0.25	0.14	0.45	0.001
miR-99b	1.64	0.59	4.55	0.494
miR-221	3.01	1.49	6.06	0.021
miR-222	1.41	0.93	2.12	0.211

	Discovery Cohort - 12 Months After Surgery vs Before			
	FC	Low CI	High CI	Adj.p-value
miR-122	0.09	0.05	0.15	3.23E-08
miR-885-5p	0.14	0.09	0.23	1.18E-07
miR-192	0.27	0.18	0.43	2.56E-05
miR-99b	1.63	1.07	2.50	0.071
miR-221	2.90	1.58	5.33	0.007
miR-222	1.48	1.03	2.13	0.080

Supplemental Table 8. Anthropometric and biochemical parameters of the validation cohort.

Values are expressed as mean ± SD. Serial adipose tissue samples were obtained from obese patients before and at various intervals after bariatric surgery i.e. 12, and 18 months after surgery. T=0 indicates timepoint before surgery, T=12 indicates timepoint 12 months after surgery, and T=18 indicates timepoint 18 months after surgery. For 12 patients, the serial measurement was obtained 12 months after surgery and for 19 patients, the serial measurement was obtained for 18 months after surgery.

	Obesity T=0	Obesity T=12	Obesity T=18
**Sample Size**	33	14	19
**Sex**	9♂, 24♀	0♂, 14♀	9♂, 10♀
**Age (years)**	36.64 ± 9.54	40.36 ± 6.44	33.89 ± 10.59
**BMI (kg/m^2)**	42.37 ± 3.85	33.96 ± 5.20	33.44 ± 5.27
**T-Chol (mg/dL)**	198.91 ± 38.89	191.07 ± 39.10	178.65 ± 32.95
**HDL-C (mg/dL)**	49.04 ± 10.40	56.96 ± 15.30	48.13 ± 12.73
**LDL-C (mg/dL)**	124.87 ± 34.93	115.05 ± 36.56	102.85 ± 33.04
**Triglycerides (mg/dL)**	123.79 ± 48.66	103.14 ± 44.56	105.00 ± 53.05
**ALT (UI/L)**	42.18 ± 33.89	17.50 ± 7.78	25.35 ± 10.59
**Insulin (µUI/L)**	19.87 ± 14.82	12.48 ± 7.63	9.55 ± 5.27
**Fasting glucose (mg/dL)**	97.94 ± 14.07	87.57 ± 7.78	97.82 ± 10.85
**CRP (mg/dL)**	1.06 ± 0.92	0.69 ± 0.64	0.29 ± 0.35
**HbA1c (%)**	5.74 ± 0.34	5.33 ± 0.15	Not Available
**HOMA-IR**	4.46 ± 2.18	2.78 ± 1.95	2.25 ± 1.39

BMI: body mass index, T-Chol: total cholesterol; HDL-C: high-density lipoprotein cholesterol, LDL-C: low-density lipoprotein cholesterol, ALT: alanine aminotransferase; CRP: C-reactive protein, HbA1c: Glycated hemoglobin, HOMA-IR: homeostatic model assessment for insulin resistance.

Supplemental Table 9: miRNA changes in the discovery and validation cohorts.

Tables showing miRNA comparisons in the discovery and validation cohort normalized to the global Ct average. The significant reductions of circulating liver-derived miRNAs (brown color) after bariatric surgery in the discovery cohort were confirmed in the validation cohort: miR-122 and miR-192 showed a significant reduction after 12 months and miR-122 and miR-885-5p showed a significant reduction after 18 months. The putative adipose tissue-derived miRNAs miR-99b, miR-221 and miR-222 (yellow color) showed no significant reduction, even after 12-18 months of bariatric surgery. FC denotes fold-change; CIs denote limits of confidence interval for FC; Adj.p-value denotes adjusted p-value or q-value.

	Discovery Cohort - 12 Months After Surgery vs Before			
	FC	Low CI	High CI	Adj.p-value
miR-122	0.08	0.04	0.14	1.05E-07
miR-885-5p	0.12	0.07	0.21	3.73E-07
miR-192	0.24	0.16	0.36	8.51E-07
miR-99b	1.43	0.94	2.19	0.133
miR-221	2.55	1.72	3.77	0.000
miR-222	1.30	1.09	1.55	0.013

	Validation Cohort - 12 Months After Surgery vs Before			
	FC	Low CI	High CI	Adj.p-value
miR-122	0.36	0.23	0.57	0.005
miR-885-5p	0.37	0.12	1.16	0.329
miR-192	0.45	0.27	0.73	0.035
miR-99b	0.97	0.59	1.59	0.889
miR-221	1.54	0.71	3.36	0.441
miR-222	0.91	0.63	1.33	0.772

	Validation Cohort - 18 Months After Surgery vs Before			
	FC	Low CI	High CI	Adj.p-value
miR-122	0.43	0.25	0.75	0.038
miR-885-5p	0.45	0.27	0.74	0.038
miR-192	0.81	0.55	1.20	0.500
miR-99b	1.07	0.71	1.19	0.783
miR-221	1.82	0.99	3.35	0.276
miR-222	0.96	0.77	1.19	0.783

Supplemental Table 10: After surgery (12 months) vs Before surgery comparison in the validation cohort normalized to the global CT average.

Detailed results of statistical comparison in the validation cohort for miRNA levels 12 months after surgery and before bariatric surgery (paired analysis), using the e-bayes algorithm of the limma package and paired analysis. Adjustment of p-values for multiple testing was conducted with the Benjamini-Hochberg algorithm. In the table below, FC denotes fold-change; CIs denote limits of confidence interval for FC; AveExpr denotes average expression; t denotes moderated t-statistic; P.Value denotes raw p-value; adj.P.Value denotes adjusted p-value or q-value and B denotes log-odds that the miRNA is differentially expressed.

	FC	Low CI	High CI	AveExpr	t	P.Value	adj.P.Val	B
**miR-122**	0.36	0.23	0.57	1.54	-4.67	0.00	0.00	0.73
**miR-192**	0.45	0.27	0.73	0.09	-3.46	0.00	0.03	-1.82
**miR-20b**	2.07	1.03	4.14	0.60	2.20	0.04	0.25	-4.30
**miR-150**	0.65	0.43	0.98	2.71	-2.19	0.04	0.25	-4.31
**miR-26a**	1.70	0.99	2.93	0.88	2.05	0.06	0.27	-4.55
**miR-885-5p**	0.37	0.12	1.16	0.21	-1.83	0.08	0.33	-4.91
**miR-29a**	0.75	0.53	1.06	0.42	-1.76	0.10	0.33	-5.03
**miR-30b**	1.45	0.89	2.36	2.07	1.60	0.13	0.37	-5.27
**miR-21**	0.63	0.33	1.21	20.88	-1.49	0.15	0.37	-5.42
**let-7b**	1.55	0.83	2.90	1.19	1.47	0.16	0.37	-5.44
**miR-145**	1.40	0.86	2.28	1.39	1.43	0.17	0.37	-5.50
**miR-223**	1.31	0.85	2.04	104.32	1.31	0.21	0.42	-5.66
**miR-148a**	0.74	0.45	1.23	1.12	-1.24	0.23	0.43	-5.74
**miR-221**	1.54	0.71	3.36	4.66	1.17	0.26	0.44	-5.82
**miR-143**	1.35	0.70	2.61	0.18	0.97	0.34	0.55	-6.02
**miR-148b**	1.36	0.67	2.78	0.28	0.91	0.37	0.56	-6.08
**miR-19b**	0.70	0.25	1.94	29.47	-0.74	0.47	0.66	-6.22
**miR-222**	0.91	0.63	1.33	3.40	-0.52	0.61	0.77	-6.36
**miR-574**	1.21	0.55	2.66	0.82	0.52	0.61	0.77	-6.36
**miR-140**	1.07	0.74	1.53	0.15	0.38	0.71	0.85	-6.42
**miR-375**	1.14	0.42	3.11	0.11	0.27	0.79	0.89	-6.46
**miR-130a**	0.95	0.55	1.64	5.77	-0.22	0.83	0.89	-6.47
**miR-27b**	1.05	0.60	1.84	2.35	0.18	0.86	0.89	-6.48
**miR-99b**	0.97	0.59	1.59	0.28	-0.14	0.89	0.89	-6.49

Supplemental Table 11: After surgery (18 months) vs Before surgery comparison in the validation cohort normalized to the global CT average.

Detailed results of statistical comparison in the validation cohort for miRNA levels 18 months after surgery and before bariatric surgery (paired analysis), using the e-bayes algorithm of the limma package and paired analysis. Adjustment of p-values for multiple testing was conducted with the Benjamini-Hochberg algorithm. In the table below, FC denotes fold-change; CIs denote limits of confidence interval for FC; AveExpr denotes average expression; t denotes moderated t-statistic; P.Value denotes raw p-value; adj.P.Value denotes adjusted p-value or q-value and B denotes log-odds that the miRNA is differentially expressed.

	FC	Low CI	High CI	AveExpr	t	P.Value	adj.P.Val	B
**miR-885-5p**	0.45	0.27	0.74	0.85	-3.30	0.00	0.04	-1.72
**miR-223**	1.52	1.16	1.99	142.95	3.23	0.00	0.04	-1.86
**miR-122**	0.43	0.25	0.75	4.31	-3.16	0.00	0.04	-2.00
**miR-145**	1.60	1.07	2.39	1.24	2.43	0.02	0.15	-3.45
**miR-221**	1.82	0.99	3.35	2.61	2.03	0.06	0.28	-4.15
**miR-324**	1.56	0.96	2.51	0.12	1.92	0.07	0.28	-4.33
**miR-574**	1.32	0.95	1.85	0.96	1.74	0.10	0.31	-4.60
**miR-140**	1.28	0.94	1.74	0.23	1.67	0.11	0.31	-4.70
**miR-143**	1.74	0.87	3.46	0.11	1.66	0.11	0.31	-4.72
**miR-27b**	1.50	0.79	2.85	1.00	1.32	0.20	0.46	-5.16
**miR-29a**	1.19	0.89	1.59	0.44	1.27	0.22	0.46	-5.22
**miR-19b**	1.17	0.90	1.51	21.34	1.26	0.22	0.46	-5.24
**miR-21**	1.25	0.85	1.84	12.12	1.19	0.25	0.47	-5.31
**miR-192**	0.81	0.55	1.20	0.16	-1.11	0.28	0.50	-5.40
**miR-148a**	0.85	0.58	1.24	0.75	-0.89	0.38	0.60	-5.60
**miR-130a**	1.17	0.81	1.67	1.20	0.89	0.38	0.60	-5.61
**miR-26a**	1.18	0.73	1.90	1.25	0.71	0.49	0.68	-5.74
**miR-30b**	1.15	0.75	1.76	2.25	0.67	0.51	0.68	-5.77
**miR-150**	0.91	0.67	1.23	3.02	-0.66	0.51	0.68	-5.77
**miR-148b**	1.27	0.50	3.24	0.06	0.52	0.61	0.76	-5.85
**miR-222**	0.96	0.77	1.19	2.12	-0.41	0.68	0.78	-5.90
**miR-99b**	1.07	0.71	1.61	0.35	0.34	0.74	0.78	-5.93
**let-7b**	0.93	0.59	1.47	1.76	-0.33	0.74	0.78	-5.93
**miR-375**	1.15	0.46	2.88	0.15	0.32	0.75	0.78	-5.94
**miR-20b**	0.95	0.56	1.63	0.76	-0.18	0.86	0.86	-5.97
